# Plant Virus–Insect Vector Interactions: Current and Potential Future Research Directions

**DOI:** 10.3390/v8110303

**Published:** 2016-11-09

**Authors:** Ralf G. Dietzgen, Krin S. Mann, Karyn N. Johnson

**Affiliations:** 1 Queensland Alliance for Agriculture and Food Innovation, The University of Queensland, St. Lucia QLD 4072, Australia; 2 Agriculture and Agri-Food Canada, Summerland Research and Development Centre, Summerland, BC V0H 1Z0, Canada; krinpreet.mann@canada.ca; 3 School of Biological Sciences, The University of Queensland, St. Lucia QLD 4072, Australia; karynj@uq.edu.au

**Keywords:** plant viruses, mosquitoes, insect vectors, virus transmission, molecular virus–insect interactions, salivary gland, insect stylet, virus control, *Wolbachia*

## Abstract

Acquisition and transmission by an insect vector is central to the infection cycle of the majority of plant pathogenic viruses. Plant viruses can interact with their insect host in a variety of ways including both non-persistent and circulative transmission; in some cases, the latter involves virus replication in cells of the insect host. Replicating viruses can also elicit both innate and specific defense responses in the insect host. A consistent feature is that the interaction of the virus with its insect host/vector requires specific molecular interactions between virus and host, commonly via proteins. Understanding the interactions between plant viruses and their insect host can underpin approaches to protect plants from infection by interfering with virus uptake and transmission. Here, we provide a perspective focused on identifying novel approaches and research directions to facilitate control of plant viruses by better understanding and targeting virus–insect molecular interactions. We also draw parallels with molecular interactions in insect vectors of animal viruses, and consider technical advances for their control that may be more broadly applicable to plant virus vectors.

## 1. Introduction—Insects as Plant Virus Vectors

The majority of plant viruses that cause disease in agricultural crops rely on biotic vectors for transmission and survival [1]. The largest class of plant virus-transmitting vectors are insects but other vectors include mites, nematodes and chytrid fungi. For a comprehensive depiction of plant virus groups and their known associated vectors see the review by Bragard and colleagues [2]. The best-characterized plant viral insect vectors are aphids, thrips, leafhoppers, planthoppers and whiteflies [2]. The different modes of viral transmission by vectors include non-persistent, semi-persistent and persistent, whereby the transmission window to disseminate the virus to a new host plant after feeding on an infected plant by the vector lasts from seconds to minutes, hours to days, or days to weeks, respectively. Non-persistent plant viruses are retained in the insect stylet. Semi-persistent viruses are internalized in the insect by binding to chitin lining the gut, but do not appear to enter tissues. Persistent viruses are taken up into and retained by insect tissues and are characterized by invading the salivary glands [3]. Persistent viruses can be further divided into circulative, non-propagative, and circulative, propagative (see the review [2] for an artistic diagram of vector–virus transmission types and characteristics). Circulative viruses must escape the insect gut and spread to neighboring organs to reach the salivary glands for transmission [2,3]. This journey is thought to involve the interactions of viral proteins with host vector proteins [3,4,5]. However, which vector components are involved and how they function to facilitate transmission is still not well understood [6,7]. 

## 2. Types of Interactions of Plant Viruses with the Insect Vector

### 2.1. Non-Persistent Transmission

**Figure 1:**
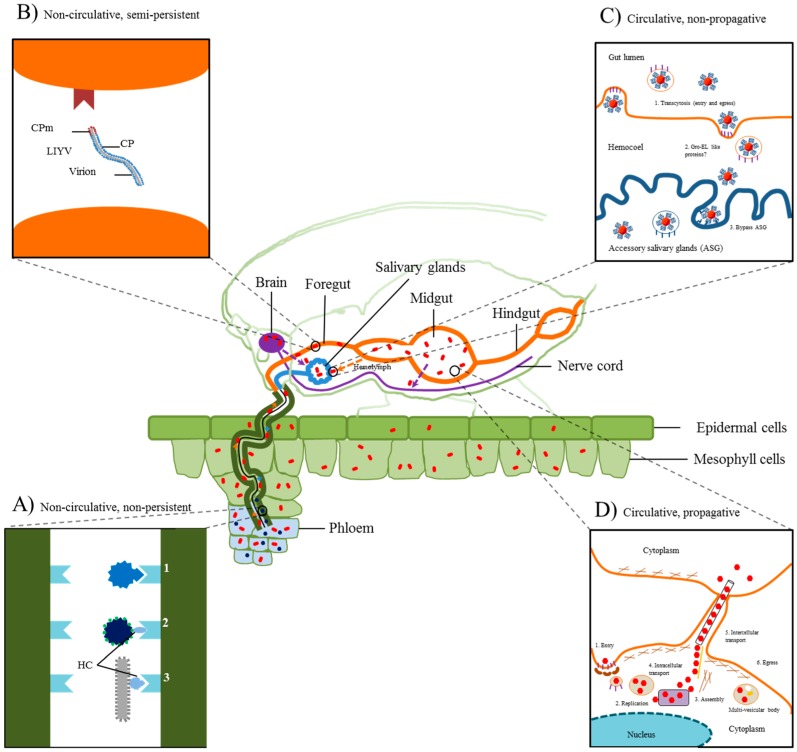
Plant virus transmission strategies in insect vectors. A viruliferous insect is shown feeding on infected phloem tissue (stylet in dark green). (**A**) Non-circulative, non-persistent viruses are retained in the distal tip of the insect stylet (small blue hexagons) through two strategies, capsid-only or helper-dependent (Inset A: Magnification of the insect stylet duct region). In capsid-only, virion attachment to the insect stylet is facilitated by a direct interaction mediated by the capsid protein (1). In helper-dependent, attachment of the virion to the stylet is accomplished using several (2; caulimoviruses) or a single non-structural protein(s) (3; potyviruses); (**B**) Non-circulative, semi-persistent viruses are retained within the insect foregut (Inset B: Magnification of insect foregut). Crinivirus (lettuce infectious yellows virus; LIYV) attachment to the insect foregut appears to be dependent on only the minor capsid protein (CPm; small brown circles); (**C**) Circulative, non-persistent viruses are non-replicating and require invasion of multiple insect organs to reach the salivary glands for transmission (Inset C: Magnification of luteovirus transmission route from the midgut to the accessory salivary glands). Following ingestion, virions are transported along the alimentary canal and transcytose across the gut (hindgut or midgut) to the hemocoel mediated by the major capsid protein (small red hexagon). However, interaction with and passing through the accessory salivary glands is thought to involve the minor read-through capsid protein (blue loops extending from small red hexagon). Bacterial endosymbionts in the hemocoel have been hypothesized to aid the transmission process for some luteoviruses and geminiviruses by secreting protective proteins; (**D**) Circulative, propagative viruses replicate and systemically invade several insect organs and tissues with the primary goal of entering the hemolymph or neuronal tissues in order to reach the salivary glands for transmission (Inset D: Magnification of the infection route of rice dwarf virus; RDV). RDV (small red hexagons) enters the midgut by endocytosis in a P2- (outer capsid protein) dependent manner. This is followed by P2-mediated release of RDV virions from endosomes to initiate viral replication and assembly. Virions are then transported cell-to-cell through viral Pns10-derived tubular structures, which bypasses the hemocoel. Modified from [1,4,5,6]. HC, helper component; CP, coat protein; ASG, accessory salivary glands.

Current evidence suggests that non-persistent plant viruses (Figure 1A) employ one of two mechanisms of transmission: capsid-only or helper-dependent [1,2,5,6]. As an example for capsid-only mechanism, cucumber mosaic virus (CMV) particles, but not isolated viral RNA, were shown to be transmissible by the aphid *Myzus persicae* [8]. Specifically, the viral coat protein (CP) [9] and conserved capsid surface domains are required to achieve efficient aphid transmission [10]. As an example for helper-dependent mechanism, cauliflower mosaic virus (CaMV) was shown to require several viral proteins, along with the virions [11,12]. CaMV P2 was shown to interact with the CP-anchored P3 and aphid stylet [6,13,14]. In addition, CaMV-induced microtubule-associated transmission bodies (TBs) were shown to reallocate key viral proteins such as P2 onto cellular microtubules upon “sensing” aphid feeding to facilitate uptake and enhance acquisition by the vector [15], and to target CaMV virions to microtubules for association with P2 [11]. High-resolution microscopy showed that CaMV P2 bound to aphid stylets and appeared to localize at the very tip of the stylet; this stylet region was proposed as a potential common foothold for non-circulative viruses [16,17]. Potyviruses encode a helper protein, helper component-proteinase (HC-Pro) [18], which is essential for virus transmission, as it facilitates virion retention in aphid stylets [19] by acting as a bridge between the potyvirus CP and aphid protein(s) in the stylet [20,21,22]. CMV 2b protein, an RNA silencing suppressor, has been shown to interfere with multiple steps of the RNA silencing pathway in plants (reviewed by [23]) and to indirectly enhance CMV transmission by promoting sustained phloem feeding and survival of the aphid vector [24]. 

### 2.2. Non-Circulative, Semi-Persistent Transmission

Semi-persistent viruses are not thought to be internalized in the insect vector gut, but instead reside in chitin-lined areas [5]. Virus acquisition from the host plant and retention in the insect involve mechanisms mediated largely by the viral CP (Figure 1B) [25]. In the case of the crinivirus lettuce infectious yellows virus (LIYV), immune fluorescent confocal microscopy has shown that virions are retained in the foregut of the whitefly vector *Bemisia tabaci,* apparently mediated by the CP [26,27]. Chen and collaborators [26] proposed that transmission of LIYV virions from the whitefly foregut is likely to occur during regurgitation rather than salivation as seen with aphids and CaMV, given that unlike aphids [16], the whitefly foregut is physically separated from its maxillary stylet and salivary duct (Figure 1B).

### 2.3. Circulative, Non-Propagative Transmission

Circulative, non-propagative viruses do not replicate in vector tissues, but traverse the insect gut, hemolymph and salivary tissue membranes to reach the salivary glands for transmission (Figure 1C) [2,28]. Luteovirids, geminiviruses and nanoviruses are vectored by phloem-feeding insects in this manner, but these vectors need to feed for extensive periods of time to facilitate efficient transmission [29]. The transcytotic dissemination pathway for this group of viruses is well characterized and is exemplified by viruses in the family *Luteoviridae* [7]. After ingestion, virions are transported along the alimentary canal where they interact with the gut epithelial surface to facilitate adsorption to hindgut or midgut cells via receptor-mediated endocytosis. Subsequently virions are delivered to the hemocoel by exocytosis and eventually pass through membranes of the salivary glands for transmission through saliva during ingestion of plant sap [29,30,31,32,33]. Acquisition and transmission of luteovirids is highly specific and appears to be facilitated by the major CP and minor CP-read-through protein (RTP) domain at different stages of the process [29,34,35,36]. CP was sufficient to transcytose virions into and out of the gut to the hemocoel while CP-RTP appeared to be required for interaction with and passing through the accessory salivary gland membranes [34,36,37]. Critical virus-associated host plant proteins that may be induced in the phloem of polerovirus-infected plants appear to be required for virus uptake and transmission by aphid vectors [38]. Interestingly, endosymbionts in the hemocoel have been hypothesized to aid the transmission process for some luteovirids and geminiviruses, but this is hotly debated [29]. Pinheiro and collaborators [28] recently conducted a critical review of the endosymbiont literature and concluded that further studies are required to clarify the proposed direct and/or indirect role of bacterial endosymbionts in plant virus transmission in different virus–vector pathosystems. These studies could potentially involve comparative studies of insects with and without their associated bacterial endosymbionts. 

Geminiviruses follow a similar circulative route [29,39,40] and also appear to require CP for transmission [41,42]. Mechanisms and components of begomovirus (family *Geminiviridae*) circulative transmission in their whitefly vector were recently reviewed [43,44]. The identification of proteins encoded by the whitefly vector and its bacterial endosymbionts that may be important in begomovirus transmission has so far met with limited success. Heat shock protein 70 (HSP70) and GroEL chaperone proteins have been implicated in transport to and stabilization in the hemolymph, respectively [43,44]. However, no strong candidate for being a begomovirus receptor has emerged thus far, despite increased next generation sequencing efforts.

### 2.4. Circulative, Propagative Transmission

Circulative, propagative viruses are characterized by replication and systemic invasion of vector insect tissues prior to transmission via salivary glands (Figure 1D) [2,4]. The infection route of the plant reovirus rice dwarf virus (RDV) in the leafhopper *Nephotettix cincticeps* [45] involves the outer CP P2 for entry into cells of the gut by endocytosis via clathrin-coated vesicles [46,47]. This is followed by P2-mediated release of RDV virions from endosomes to initiate viral replication, assembly and association with RDV-induced tubular structures [48,49]. RDV non-structural Pns10 protein forms cell-to-cell movement related-tubules in plants that are also critical for intracellular spread in the leafhopper vector. Virions move through these tubular structures along actin-based microvilli and through gut muscle tissue in the alimentary canal to facilitate spread throughout the insect body including the salivary glands [50]. RDV Pns10 was also shown to specifically interact with the cytoplasmic actin of *N. cincticeps* (main vector) but not *Recilia dorsalis* (inefficient leafhopper vector) suggesting that actin was both important for transmission and virus–vector specificity [51]. The reovirus southern rice black-streaked dwarf virus (SRBSDV) escapes the initially infected midgut epithelium using tubules to cross the basal lamina barrier in the intestine to facilitate rapid dissemination in the planthopper vector *Sogatella furcifera* [52,53]. This speeds up the invasion process as demonstrated by a shorter latent period observed for SRBSDV (6–9 days) in comparison to RDV (14–21 days) [54,55]. SRBSDV tubules are composed of the viral non-structural protein P7-1 that directly interacts with actin. Reoviruses appear to use similar tubule-mediated mechanisms for intercellular movement in both their plant and insect hosts.

Tomato spotted wilt virus (TSWV) and other tospoviruses also generate tubules for viral movement in plants [56]. However, no tubular structures have been observed during the infection process of the thrips vector *Frankliniella occidentalis,* even though in insect cell lines tubules can be generated from the non-structural movement protein NSm [57]. The route of TSWV dissemination and virus–vector interactions in thrips is well characterized [1]. Infection of salivary glands likely occurs as a result of TSWV accumulating in the visceral muscles of the midgut and foregut, and the ligaments that connect the midgut to the salivary glands after leaving the midgut epithelial cells [58,59]. Numerous studies have demonstrated the importance of TSWV surface glycoproteins Gn and Gc in facilitating entry into the thrips midgut [60,61,62]. A soluble form of Gn protein specifically bound to thrips midgut epithelial cells in vivo and competitively reduced TSWV binding [63]. Gc protein is thought to facilitate TSWV entry into thrips tissues, but this remains to be functionally demonstrated [64,65]. Despite these findings, little is known about host factors or receptors that may be required for acquisition of TSWV by thrips. However, recent availability of thrips genome, transcriptome and proteome data can be expected to assist identification of thrips factors involved in successful tospovirus acquisition and transmission [66,67].

Tenuiviruses are transmitted by planthoppers and leafhoppers and their vector dissemination route includes diverse organs [68,69]. Different tenuiviruses show distinct tissue tropism. For instance, rice grassy stunt virus dissemination in the planthopper *Nilaparvata lugens* involves invasion of the principal and accessory salivary glands but does not involve entry into neural tissues or ovarioles [69], while rice stripe virus (RSV) is found in the ovarioles and only the principal salivary glands in the planthopper *Laodelphax striatellus* [70,71]. Interestingly, although tenuiviruses are non-enveloped, they appear to encode glycoproteins resembling the Gn and Gc proteins of tospoviruses. Both tenuivirus glycoproteins exclusively localize to the endoplasmic reticulum (ER) in insect cells [72] and they are thought to aid virus uptake during feeding by acting as helper components similar to those described for non-circulative viruses [73]. Tenuivirus non-structural protein(s) (NS) appear to promote spread within the vector and transovarial transmission from infected females to the offspring. NS4 protein is required for RSV spread through visceral muscle tissues to the salivary glands in the planthopper vector by direct interaction between NS4-induced cytoplasmic inclusions and RSV filamentous RNP particles [71]. RSV invasion of ovarioles was shown to be dependent on the host protein vitellogenin (Vg) in *L. striatellus* and involves Vg interaction with RSV major nucleocapsid protein pc3 [74]. 

In the *Rhabdoviridae*, monopartite cyto- and nucleorhabdoviruses with known vectors are transmitted in a persistent, circulative-propagative manner by aphids, planthoppers or leafhoppers [4,75,76]. Midgut epithelial cells of hemipteran insects are lined with muscle tissues that control the motion of the foregut and midgut, and are linked to nervous tissue through a network of ganglia [77]. Plant rhabdoviruses acquired during sap feeding can use these connections as an alternative route to the salivary glands. Based on confocal microscopy and immunofluorescence studies, maize mosaic virus (MMV) acquired by the planthopper vector *Peregrinus maidis* appears to breach the transmission barriers in *P. maidis* by spreading from the midgut to the anterior diverticulum and esophagus, a region that spans the compound ganglia and salivary glands, and then invades neuronal tissues including brain, compound ganglion and compound eye cells to gain access to the salivary glands [78]. Plant rhabdovirus glycoprotein (G) spikes on the surface of virions are predicted to interact with receptors in the midgut allowing virions to enter epithelial cells by endocytosis [4,75], but little is known about the molecular interactions of plant rhabdoviruses with their insect vectors and G protein receptors remain to be identified [76]. 

## 3. Critical Steps in Virus–Insect Interactions as Potential Control Targets

Insect vectors play a crucial role in determining the plant host range of a virus. Competency for virus transmission is determined by genetic components and specific molecular interactions of virus and vector, and potentially host plant components taken up during feeding. Better knowledge of these interactions and mechanisms of transmission will be essential for developing more effective control measures in future. Currently available strategies to interfere with virus transmission by vectors include host genetic resistance to virus and/or insect, insecticides and integrated pest management [2]. Critical steps in virus–vector interactions can be expected to vary depending on virus transmission mode that may involve the feeding mouthparts (stylet-borne), foregut (semi-persistent) or virus uptake into and circulation through the insect body (persistent). Interestingly, infection of the foregut-borne, semi-persistently leafhopper-transmitted maize chlorotic dwarf virus led to a significant induction of the insect’s innate immune system, similar to that induced by the persistent-propagative maize fine streak virus (MFSV), indicating potentially conserved responses and targets to disrupt vector-mediated plant virus transmission [79].

An excellent recent review discusses potential avenues to disrupt or interfere with insect transmission of plant viruses [80]. The main steps considered were virus acquisition from the host plant and transport and delivery to a new host. For the latter, viral CP or surface glycoproteins are required to interact with receptors or other recognition molecules such as viral attachment proteins in the vector and thereby determine vector specificity. Such defined steps likely represent good targets to block virus uptake or disrupt transmission. Emerging translational technologies aimed at disrupting essential virus–vector interactions include blocking virus entry into the vector using a competing viral protein or interfering with virus-interacting proteins in vector tissues. Alternative technologies that can kill plant virus vector insects include delivery of either fusions of viral CP with a toxin or insect gut-binding peptide-toxin fusions [80]. 

### 3.1. Stylet-Borne Viruses

Emerging evidence suggests that non-circulative, “stylet-borne” virus transmission involves more than passive adhesion to the sucking-feeding mouthparts, and instead results from specific molecular interactions between virus, plant and vector. 

The concept of “transmission activation” has been proposed, based on the immediate intracellular response of CaMV to the presence of the aphid vector on the plant [15]. Aphid probing was shown to lead to disintegration of so-called transmission bodies to form mixed networks for enhanced virus uptake. Insect probing is predicted to trigger eliciting events in plant cells involving mechanical and unknown chemical components that could be of plant and vector origin. Future exploration of the molecular and cellular basis of this interaction may reveal novel transmission control options. It should be investigated if this phenomenon occurs more generally in non-circulative plant viruses [81].

### 3.2. Persistent Viruses

During the infection process of persistent, propagative viruses such as rhabdoviruses (family *Rhabdoviridae*), tospoviruses (family *Bunyaviridae*), tenuiviruses and reoviruses (family *Reoviridae*), viruses encounter multiple barriers to acquisition, replication, intercellular movement, cell escape and host plant inoculation [50,82,83,84]. Virus and insect proteins thought to be involved in overcoming these barriers [3,83] are only beginning to be identified. Rhabdovirus and tospovirus surface glycoproteins are required for virus entry into vector cells (genetic determinants of transmissibility), but their counterpart receptors in the insect gut have yet to be identified [62,82,84]. Identification of receptor-like determinants in the insect vector that viruses bind to would constitute a major breakthrough since they could become novel targets to control virus acquisition. Towards this goal, the first receptor for a circulative plant virus was recently identified in the pea aphid. Membrane alanyl aminopetidase N was shown to act as a receptor for pea enation mosaic virus (PEMV) CP in the insect gut [85]. 

Plant reovirus infections of their vectors lead to the generation of tubules composed of viral non-structural proteins, which interact with actin. These tubules facilitate rapid virus dissemination from the midgut across internal barriers to other insect tissues. Assembly of SRBSDV tubules that are composed of the viral non-structural protein P7-1 can be prevented by targeting the viral genome segment that encodes this protein using RNA interference (RNAi) leading to the inhibition of internal virus spread [53]. Anti-viral RNAi technologies have great potential for plant virus control, in particular when delivered in transgenic plants.

Replicating plant viruses have been shown to activate the immune response of their insect vectors. This was first demonstrated for TSWV through up-regulation of thrips transcripts for antimicrobial peptides, pathogen recognition and innate immune system activation [86]. Immune response genes in the transcriptome of *Graminella nigrifrons*, the leafhopper vector of the nucleorhabdovirus MFSV, were shown to be differentially expressed when insects fed on virus-infected leaves [87,88]. The gut transcriptome of the corn planthopper *P. maidis* that transmits MMV shows innate immunity and RNAi pathway genes upregulated [89]. Persistent infection of a plant reovirus in its leafhopper vector led to conserved siRNA antiviral response that appeared to modulate persistent infection [90]. A better understanding of insect vector immune response may assist future development of control strategies.

The salivary gland of transmission-competent sucking vector insects deserves special attention. Saliva plays a crucial role in insect feeding behavior and virus transmission [91]. In thrips, the primary site of TSWV infections shifts from the midgut and tubular salivary glands in larvae to primary salivary glands in adult vectors [92]. This change in tissue tropism during insect development leads to virus accumulation ready for transmission. It is also feasible that insect saliva composition may affect virus stability and ability to establish infection in the plant, but this remains to be investigated.

### 3.3. Plant Virus Effects on Vector Behaviour

Plant viruses can modify insect vector behavior directly, and indirectly by manipulating plant hosts, leading to enhanced transmission efficiency and spread [93]. Directly modified feeding behaviors that increase virus transmission have been reported for persistently TSWV-infected *Frankliniella occidentalis* thrips [94]. The non-persistently aphid-transmitted CMV was similarly found to induce specific changes in the host plant such as suppression of the jasmonic acid signaling pathway that affected aphid behavior leading to enhanced performance, which appear to be indirectly linked to the viral 2b protein [24,95]. The geminivirus tomato yellow leaf curl virus (TYLCV) was shown to manipulate feeding behaviors of whiteflies to increase virus transmission [96]. Further investigations of the processes underlying these behavioral changes in the insect may elucidate which factors are involved.

Virus-induced biochemical and physiological changes in the host-plant have been shown to influence vector insect host preference [97]. Virus-infected plants can emit volatiles that make them more attractive to insect feeding [98,99]. Virus-infected plants generally appear superior quality hosts for vectors compared to uninfected plants, thus enhancing vector life history and virus spread [98]. However, examples of reduced host plant quality leading to rapid vector dispersal have also been reported [97]. Luteovirid acquisition by aphids appears to alter host selection behavior to prefer uninfected plants while non-viruliferous aphids tend to prefer virus-infected plants, thereby promoting both virus acquisition and transmission [99,100]. Similar virus effects on host preferences of the vector were observed for reovirus-infected and virus-free planthoppers [101]. The nuclear inclusion a (NIa) protease protein of turnip mosaic potyvirus has been implicated in manipulating host plant physiology to attract aphid vectors and to promote their reproduction [102]. 

## 4. What Can We Learn from Research on Virus Interactions with Vectors of Animal Viruses?

While the processes involved in transmission of viruses directly from plant to plant or animal to animal vary substantially, viruses of both plants and vertebrates that are vectored by insects have many parallels in their interactions with their insect host. Insects that vector viruses are mainly hemipteran sap-feeders for plants and mainly dipteran blood-feeders (primarily mosquitoes) for vertebrates. In both cases the virus is acquired during feeding on the non-insect host and then is transferred to a naïve non-insect host again via feeding. The interaction with the insect host for vertebrate viruses is considered either biological (the virus replicates in the vector) or mechanical (not involving replication) [103]. As discussed above, non-circulative and circulative non- propagative transmission of plant viruses are common and involve specific interactions in the insect host. In contrast, for vertebrates there is evidence that mechanical transmission can occur following a blood feed in a non-specific interaction. However, relatively few studies have focused on identifying the mechanisms that underlie mechanical transmission [104,105,106], raising the possibility that some specificity in acquisition and retention occurs but is yet to be systematically documented [107]. Stronger parallels lie in biological and propagative transmission, which represent different terminology to describe similar interactions with the vector host. Thus research into harnessing biological infection of the insect vector to control disease transmission to vertebrate animals may provide insights for similar approaches to control transmission of plant viruses and vice versa.

A major focus of insect vector transmitted vertebrate viruses is the mosquito-borne viruses that cause significant human disease. These include viruses from three RNA virus families (*Flaviviridae*, *Togaviridae* and *Bunyaviridae*) with *Aedes* and *Culex* species mosquitoes transmitting the majority of these [108]. For many of these highly pathogenic viruses, which include Dengue virus (DENV) and Chikungunya virus, the infection cycle alternates between the insect and human hosts. Control of the insect vector remains the major point of control of virus infection and this is heavily reliant on insecticides. Since current approaches are not sufficient to control virus spread and insecticide resistance is rising [109,110], there is a current emphasis on insect vector–virus interactions and novel insect control measures. 

Transmission of viruses by mosquitoes can be decreased either by vector population reduction or increasing the ratio of mosquitoes refractory to virus infection. Here we focus on the control of vector competency. Within natural populations there is variation in the susceptibility of mosquitoes to a given pathogenic virus, but there are also clear variations in the vector–virus interactions that are specific to both host and virus genotypes (genotype × genotype interactions) [111]. Understanding what allows a mosquito to be refractory may help develop strategies for virus control, with the strongest candidates being those causing decreases in mosquito susceptibility across broad virus genotypes. Similarly genotype × genotype interactions are known to be important in interactions of plant viruses and their vectors [83,112,113,114].

Mosquito-borne viruses generally cycle between infection of the vertebrate and insect hosts. Many mosquito vectors require a vertebrate blood meal to reproduce. Following mosquito acquisition of virus via feeding on an infected vertebrate host, the virus enters and replicates in the cells of the gut epithelium, spreads via the hemocoel to other tissues and organs, culminating in transport to and infection of the salivary glands. The virus then escapes the salivary glands and is transmitted in the saliva when the infected insect feeds on a new vertebrate host (reviewed in [115]). Following the extrinsic incubation period (the period between acquisition and onset of transmission), the virus can persist in the mosquito tissues and transmission by the infected mosquito continues throughout the insect’s lifespan. 

Efficient virus transmission is a balance between virus infection and insect host health. While mosquito-borne viruses are commonly pathogenic to their vertebrate host, biological infection of the insect host has few major pathogenic impacts on the insect host, allowing efficient transmission of the virus by the insect vector [116]. The virus can however have some impact on the mosquito fitness and recent studies indicate that there is an intricate interaction between virus and vector, which can influence vector competence (reviewed in [117]). A number of factors can contribute to the control of virus infection within the mosquito host, which in turn impact virus transmission to the vertebrate host. In addition to the mosquito infection barriers [115,117], the mosquito innate immune system includes the intrinsic RNAi pathway and several inducible systems, including Toll, immune deficiency (IMD) and Janus kinase/signal transducers and activators of transcription (JAK-STAT) pathways [116,118,119]. The *Drosophila* model system has been used to identify many of these antiviral immune pathways [120,121], but interestingly many of the *Drosophila* viruses can be highly pathogenic to their host. Persistent infection of *Drosophila* can be established following integration of DNA copies of fragments of an RNA virus genome, created by reverse transcription by a retrotransposon reverse transcriptase. These integrated sequences then act as the templates for initiation of the RNAi response against the virus [122]. Thus understanding the fine balance between virus and host response that leads to persistence may facilitate approaches to interfere with mosquito transmission of viruses. 

The mosquito microbiome also plays a role in the outcome of virus infection. The mosquito gut microbiome is diverse and can influence mosquito vector competence (reviewed in [119,123,124]). Perturbation of the bacterial microbiota can influence virus infection in mosquitoes. Decreasing the bacterial community with antibiotics increased virus replication, whereas re-introduction of bacteria species into aseptic mosquitoes reduced susceptibility of the midgut tissue to virus infection [125,126]. Interestingly, some bacterial species can also enhance mosquito susceptibility to arboviruses and in one case virus infection is dependent on the microbiota [127,128,129]. There is evidence that bacteria can regulate virus infection via either immune stimulation or production of secondary metabolites with antiviral activity [130,131]. The potential to harness the power of the microbiota to control arboviruses remains to be elucidated.

Bacteria identified in non-gut tissue, such as intracellular bacteria *Wolbachia* and *Spiroplasma*, are commonly identified in the mosquito microbiota. While *Spiroplasma* can be pathogenic in mosquitoes, their role in mosquito competence to vector viruses remains to be determined [132,133,134]. In contrast, *Wolbachia pipientis* has antiviral effects in both naturally infected and experimentally infected insects. *Wolbachia* is an endosymbiotic bacteria commonly found in cells of the reproductive tissues, salivary glands, Malpighian tubules, head and muscle [135]. *Wolbachia* is maternally inherited and is well known for its ability to invade invertebrate populations via modification of the reproductive system of the host insect [135]. In *Drosophila*, flies naturally infected with *Wolbachia* can be protected from RNA virus infection ([136,137,138], reviewed in [139]). While strong antiviral effects are yet to be identified in mosquitoes naturally infected with *Wolbachia* (reviewed in [140]), artificial infection of mosquitoes with *Wolbachia* leads to significant disruption of RNA virus replication and transmission ([141,142,143], reviewed in [144]). The dramatic antiviral effect of *Wolbachia* in artificially infected mosquitoes, together with the reproductive genetic drive are being harnessed in field trials using *Wolbachia*-infected *Aedes aegypti* mosquitoes to control DENV transmission [145,146].

The advent of the ‘omics’ age has identified a plethora of sequences of important arboviruses within vector mosquitoes. In a number of cases these virus sequences have been matched to infectious viruses, which have been shown to have a host range restricted to insects, and are termed insect specific viruses (ISVs) [147]. ISVs are defined by the ability to replicate in the insect host/insect cell culture, but not in vertebrate hosts/cell culture. ISVs have been described from virus families including *Bunyaviridae*, *Flaviviridae*, *Rhabdoviridae*, *Togaviridae*, *Reoviridae*, *Mesoniviridae* and the newly recognized taxon Negeviruses (positive-sense RNA viruses) [147]. ISVs have the potential to interfere with vector competence of mosquitoes for arboviruses either through super-infection exclusion or by immune priming. Although relatively little is known about the impact of ISVs on arboviral infections in mosquitoes, studies have utilized the insect specific flaviviruses (ISFs) (reviewed in [148]). There is some evidence that prior infection of *Culex* mosquitoes with *Culex* flavivirus reduced susceptibility to West Nile virus (WNV) infection [149] and in vitro studies in C6/36 ISF-infected cells have in some cases similarly demonstrated reduced replication of several arboviruses [150,151]. The generality of the impact of ISVs on arbovirus vector competence remains to be investigated and more research is required to understand these interactions before considering their potential utility in control measures.

In addition to the bacterial and viral microbiota, mosquitoes are commonly associated with yeast and fungi (reviewed in [152]). Comparatively little is known of the interactions of these microbes with viruses in mosquitoes, however they may be useful for para-transgenic approaches to control virus infection [152].

## 5. Significant Technological Advances in Vertebrate Virus—Insect Vector Control

Strategies harnessing features of the microbiome–virus–vector interactions are most developed for *Wolbachia* (reviewed in [145,146]). Several mosquito species stably transfected with *Wolbachia* in the laboratory are refractory to transmission of human pathogenic viruses including DENV, Chikungunya virus, WNV, yellow fever virus (reviewed in [140,144]) and Zika virus [153]). This feature has led to development of a DENV control strategy, which involves release of *Wolbachia*- infected mosquitoes into areas with endemic DENV. The approach relies on the ability of *Wolbachia* to invade mosquito populations via reproductive manipulations, replacing uninfected mosquitoes with *Wolbachia*-infected mosquitoes. Stable population replacement has been demonstrated in field trials in Australia [154,155]. However DENV is not endemic in Australia, so other trials are underway to determine the efficacy of the approach in dengue endemic areas [156]. 

Other microbes of mosquitoes have the potential as platforms for control of arboviruses. Recently, bacteria have been utilized as a vehicle to generate and deliver double-stranded RNA (dsRNA) to mosquitoes to manipulate gene expression, and adaptation of this approach to manipulate arbovirus infection is an interesting (but yet unrealized) para-transgenic approach [157,158]. Other para-transgenic approaches could be developed using the diverse microbiota of the mosquito, although to date these approaches have focused mainly on control of malarial parasites. For viruses the para-transgenic or transgenic approach may utilize the building knowledge of natural antiviral responses and physiological blocks to virus infection (for review [148]).

Recently the exciting CRISPR/Cas9 genome editing system has been adapted to introduce refractory genes into the Asian malaria vector *Anopheles stephensi* [159]. An important modification of the CRISPR/Cas9 approach was to include a genetic drive technology (termed MCR—mutagenic chain reaction) [160], which will enable a high frequency of germ-line gene conversion in progeny derived from transgenic males. The MCR cassette includes the Cas9 nuclease open reading frame (ORF) under control of a germline-specific promoter and a gene encoding a guide-RNA (gRNA) under the control of the U6 promoter. gRNA-driven site specific integration of the MCR cassette including the refractory genes into the genome eliminates the integration site in the process. Expression of Cas9 and the gRNA in the germline catalyzes a double-strand break in the DNA at the same locus in the sister chromosome. Homologous repair using the integrated MCR cassette as a template, results in the germline cell genome being homozygous for the MCR cassette and refractory gene. This allows the introduced gene to spread through a population at much higher rates than by Mendelian genetics. This technology could be utilized to drive antiviral genes through a virus vector population [161]. In theory this could also be applied to vectors of persistent plant viruses, and adaptation of the CRISPR/Cas9 technology to pea aphids is currently being explored (personal communication, Jennifer Brisson), although caution should be exercised in development of gene drives based on this system [161]. Vector populations may also be suppressed through virus infection as reviewed by [1].

## 6. Experimental Approaches to Facilitate Further Insights and Translational Research

Application of molecular technologies to the investigation of virus–vector interactions can be expected to provide deeper insights in the future. Some of these may be transposed from advances in the understanding of virus-mosquito interactions, although there are both parallels and differences between plant and animal/human systems. The bacterial endosymbiont *Wolbachia* has antiviral effects on virus transmission in mosquitoes [140,144] but this has not yet been studied in plant virus vectors. *Wolbachia* is a common endosymbiont of insects and is predicted to be present in over 40% of arthropod species [162]. Although *Wolbachia* has not been identified in the vast majority of aphid species, *Wolbachia* is known to be present in at least some plant feeding insect vectors including the whitefly [135,163,164,165]. Using a metagenomic approach, *Wolbachia* was recently shown to play a role in the supply of essential nutrients to the banana aphid [166]. Antibiotic removal of these symbionts may have detrimental effects on aphid vector survival. The antiviral effects of *Wolbachia* in the mosquito are strongest following experimental transfer of the bacteria into a new host [140], the possibility that experimental introduction of *Wolbachia* into plant virus vectors may interfere with virus replication or transmission is yet to be examined. Other parts of the vector insect microbiome may also have an effect and one might imagine use of inhibitory microbes to block plant virus transmission. Insect-associated bacteria could also potentially be used to deliver dsRNA to knockdown vector genes that are essential for insect survival or that code for virus receptors. It may also be possible to use engineered insect or plant viruses to deliver insect genome editing tools based on programmable RNA-guided nucleases such as zinc-finger nucleases or CRISPR/Cas9. Interestingly, although potential virus receptors for bunyavirus early interactions with host cells were demonstrated in vertebrates, none appear to have been identified in their arthropod vectors [167]. On the other hand, an unknown 50 kD protein expressed in the midgut of thrips larvae was identified as receptor for TSWV glycoproteins [167,168] and alanyl aminopeptidase N has been identified as a receptor for PEMV CP in the pea aphid [85]; clearly further studies using various ‘omics’ approaches will be required to identify and validate virus receptors in arthropod vectors of vertebrate and plant viruses. 

The use of green fluorescent protein tagged viruses will aid visualization of virus uptake, tissue tropism, intercellular movement in the vector, and transmission. This will require the use of reverse genetics systems to generate infectious virus clones that are available for many DNA and positive-sense RNA viruses, but are lacking for negative-sense RNA viruses such as TSWV. Only recently has the first reverse genetics system been developed for a plant rhabdovirus, which may serve as a model to develop such tools for other negative-sense RNA viruses [169]. Mutational analysis of infectious clones will also allow identification of gene function and interactions of plant viruses with host proteins in the vector insect or insect cell lines [76]. Establishment of permanent vector cell lines for more virus–vector systems will facilitate more detailed studies of virus replication and molecular interactions. 

Immunofluorescence confocal laser scanning microscopy has been used to identify a neurotropic route for MMV in its planthopper vector [78] and could be more broadly applied to examine other propagative virus–vector tissue interactions. Increased use of three-dimensional imaging techniques can be expected to provide a better understanding of structural and dynamic aspects of viral infection [170]. 3D imaging in combination with live cell imaging and correlative microscopy provide powerful tools to visually explore viral entry, movement between tissues, propagation and egress. 

RNAi is increasingly used for functional genomic studies of vector insects including leafhoppers, aphids and thrips, and to knock down insect proteins that interact with virus proteins [51]. These analyses are aided by emerging insect vector genome and transcriptome resources (http://www.aphidbase.com), including the genomes of the pea aphid (*Acyrthosiphon pisum*) and green peach aphid (*M. persicae*). Draft genomes for the western flower thrips (*F. occidentalis*) and brown planthopper (*N. lugens*) are also now available and others are in progress through the i5K project (http://arthropodgenomes.org/wiki/i5K). Transcriptome analysis of transmission-competent vectors using RNA sequencing and quantitative RT-PCR has been used to identify insect defense response genes and pinpoint expression in relevant tissues like insect gut, salivary gland and nervous cell tissues [79,87,89].

A foundational toolbox of sequence-based resources has been developed to elucidate thrips gene function and understand thrips-plant interactions and tospovirus transmission [83] that may serve as a good model for other virus–vector systems. RNAi has been shown to be effective in thrips and a microinjection system to deliver dsRNA has been developed [171]. RNAi-based strategies should enable exploration of thrips gene function with regard to virus–vector interactions aimed at disrupting tospovirus transmission. The CRISPR/Cas9 system may serve as a tool for thrips genome editing [172]. A draft genome, and transcriptome and proteome data for whole body and different developmental stages of *F. occidentalis* are available, and differentially expressed (DE) genes in response to TSWV infection including antiviral defense are being annotated [66]. Global gene expression in response to TSWV infection identified DE genes in cellular processes and innate immune response [173]. Primary salivary gland transcriptome data identified genes involved in detoxification and inhibition of plant defense responses, and genes that may play a role in the extra-oral digestion of plant tissues and sugars [91]. 

The binding and functional properties of virus acquisition determinants could be exploited in translational research approaches for improved disease control [1]. This could entail the blocking of virus acquisition or transmission as shown for tospoviruses using recombinant viral attachment proteins [174]. Similarly, a small aphid gut binding peptide was shown to interfere with enamovirus uptake from pea aphid gut into the hemocoel; the peptide also bound to gut epithelia of two other aphid species suggesting potential broader applicability [175]. Aphid vector genomic and proteomic analysis identified cyclophilin proteins as potential novel control targets based on their involvement in luteovirus transmission [176]. Vector populations may be specifically controlled by delivery of insecticidal molecules to the insect hemolymph, such as fusing a toxin to the CP of a luteovirus [177]. 

## 7. Conclusions

Protein–protein interactions between plant viruses and their insect vectors are an essential molecular interface that determines acquisition from infected host plants and transmission to new hosts. The apparent specificity of these interactions in both non-persistent and persistent plant virus transmission opens avenues for interference and control of the vector populations as well as virus transmission. While several viral interacting proteins are known, insect vector receptor-like molecules are only beginning to be identified. Increasing application of ‘omics’ technologies has the potential to contribute significantly to the identification and validation of virus-interacting proteins in the vector, as well as, a better understanding of innate immune responses to viral infection. Much knowledge can be gained from the application of emerging technologies including reverse genetics systems applied in insects and insect cells, high resolution imaging techniques, functional genomics and gene editing. Direct and indirect effects on vector behavior in relation to the infected plant host is another important research area impacting on virus acquisition and dissemination that once better understood, may lend itself to adoption of novel control strategies.
